# Seven-CpG-based prognostic signature coupled with gene expression predicts survival of oral squamous cell carcinoma

**DOI:** 10.1186/s13148-017-0392-9

**Published:** 2017-08-24

**Authors:** Sipeng Shen, Guanrong Wang, Qianwen Shi, Ruyang Zhang, Yang Zhao, Yongyue Wei, Feng Chen, David C. Christiani

**Affiliations:** 1 Department of Biostatistics, School of Public Health, Nanjing Medical University, Nanjing, China; 2 China International Cooperation Center of Environment and Human Health, Nanjing Medical University, Nanjing, China; 3 National Health and Family Planning Commission Contraceptives Adverse Reaction Surveillance Center, Jiangsu Institute of Planned Parenthood Research, Nanjing, China; 4 Department of Environmental Health, Harvard School of Public Health, Boston, MA USA; 5 Ministry of Education Key Laboratory for Modern Toxicology, School of Public Health, Nanjing Medical University, Nanjing, China; 6 101 Longmian Avenue, Nanjing, Jiangsu 211136 China

**Keywords:** Oral squamous cell carcinoma, Overall survival, Methylation, Gene expression, Prognostic signature

## Abstract

**Background:**

DNA methylation has started a recent revolution in genomics biology by identifying key biomarkers for multiple cancers, including oral squamous cell carcinoma (OSCC), the most common head and neck squamous cell carcinoma.

**Methods:**

A multi-stage screening strategy was used to identify DNA-methylation-based signatures for OSCC prognosis. We used The Cancer Genome Atlas (TCGA) data as training set which were validated in two independent datasets from Gene Expression Omnibus (GEO). The correlation between DNA methylation and corresponding gene expression and the prognostic value of the gene expression were explored as well.

**Results:**

The seven DNA methylation CpG sites were identified which were significantly associated with OSCC overall survival. Prognostic signature, a weighted linear combination of the seven CpG sites, successfully distinguished the overall survival of OSCC patients and had a moderate predictive ability for survival [training set: hazard ratio (HR) = 3.23, *P* = 5.52 × 10^−10^, area under the curve (AUC) = 0.76; validation set 1: HR = 2.79, *P* = 0.010, AUC = 0.67; validation set 2: HR = 3.69, *P* = 0.011, AUC = 0.66]. Stratification analysis by human papillomavirus status, clinical stage, age, gender, smoking status, and grade retained statistical significance. Expression of genes corresponding to candidate CpG sites (*AJAP1*, *SHANK2*, *FOXA2*, *MT1A*, *ZNF570*, *HOXC4,* and *HOXB4*) was also significantly associated with patient’s survival. Signature integrating of DNA methylation, gene expression, and clinical information showed a superior ability for prognostic prediction (AUC = 0.78).

**Conclusion:**

Prognostic signature integrated of DNA methylation, gene expression, and clinical information provides a better prognostic prediction value for OSCC patients than that with clinical information only.

**Electronic supplementary material:**

The online version of this article (doi:10.1186/s13148-017-0392-9) contains supplementary material, which is available to authorized users.

## Background

Oral squamous cell carcinoma (OSCC) is the most common head and neck squamous cell carcinoma (HNSCC), affecting approximately 48,000 individuals and causing 9500 deaths in the USA in 2016 [1]. The overall 5-year survival rate for OSCC is around 60% [2] and has only improved modestly over the past two decades despite considerable improvements in the treatment of OSCC [3, 4]. This can be attributed to limited understanding of OSCC carcinogenesis, development, progression, invasion, and metastasis [5], which sharply delays early diagnosis. Therefore, identification of molecular changes in significant oncogenes or tumor suppressor genes associated with OSCC will help improve survival prediction and early treatment [6, 7].

Epigenetic changes are inheritable and reversible, affecting the spatial conformation of DNA and its transcriptional activity [8]. DNA methylation changes may influence gene expression and interact with various positive and negative feedback mechanisms [9]. Therefore, aberrant methylation CpG sites have been considered potential prognostic factors not only in OSCC [10] but also in other cancers as well [11–13].

Previous studies have reported survival-related OSCC biomarkers at different omics levels, including somatic mutations [14], gene expression [15], miRNAs [16], and proteins [17]. Methylation markers have also been reported [18, 19]. However, these studies have relatively small sample sizes and are limited to a single epigenetic level. Therefore, more attention should be given to the relationship between methylation and gene expression [20].

In this study, we investigated the prognostic value of methylation biomarkers for OSCC overall survival. We generated a prognostic model using data from The Cancer Genome Atlas (TCGA), which are now continually hosted at the Genomics Data Commons (GDC), and validated our classifier using two independent external validation sets from Gene Expression Omnibus (GEO).

## Methods

### Study population

The training set including 313 OSCC cases were downloaded from the TCGA data portal accessed on March, 2016. Tumor sites of oral cavity, oral tongue, buccal mucosa, lip, alveolar ridge, hard palate, and floor of mouth were included. Patients were diagnosed during 1992–2013, and those with missing follow-up information were excluded. Of them, 32 OSCC patients had both tumor and adjacent non-tumor tissue samples, which was used as the discovery set to identify differential methylation CpG sites.

Clinical and DNA methylation data for validation set 1 and set 2 were obtained from GEO [accession number: GSE52793 [19] and GSE75537 [21]]. One sample in the validation set 2 were removed due to missing survival information. Clinical information was described in Table 1.

**Table 1:** Demographic and clinical characteristics of OSCC patients

Characteristic	Training set (*N* = 313)	Validation set 1^a^ (*N* = 82)	Validation set 2 (*N* = 53)
Censor rate	66.4%	71.9%	71.6%
Age, median years (range)	61.0 (19–90)	58.0 (23–85)	45.0 (28–79)
Gender, *n* (%)			
Male	206 (65.8)	36 (43.9)	11 (20.7)
Female	107 (34.2)	46 (56.1)	42 (79.3)
Smoking status, *n* (%)			
Never	87 (27.8)	30 (36.6)	–
Current/former	217 (69.3)	44 (53.7)	–
NA	9 (2.9)	8 (9.8)	–
Race, *n* (%)			
White	272 (86.9)	79 (96.3)	–
Other	31 (9.9)	3 (3.7)	–
NA	10 (3.2)	0 (0)	–
HPV status, *n* (%)			
Positive	14 (4.5)	9 (11.0)	22 (41.5)
Negative	176 (56.2)	64 (78.0)	16 (30.2)
NA	123 (39.3)	9 (11.0)	15 (28.3)
TNM stage, *n* (%)			
Early (I–II)	88 (28.1)	48 (58.5)	18 (34.0)
Advanced (III–IV)	218 (69.6)	34 (41.5)	35 (66.0)
NA	7 (2.2)	0 (0)	0 (0)

*NA* not available

^a^Baseline information of validation set 1 is collected from [19]

### Preprocessing of DNA methylation chip data

Genome-wide DNA methylation of the training set was profiled using Illumina Infinium HumanMethylation450 BeadChips Assay. Raw data (level 1 data from TCGA) were processed using R package *minfi* version 1.20.0 [22]. Background subtraction, quantile normalization, and quality control were performed subsequently. Low-quality probes were removed if they met the following criteria: (i) failed detection (*P* > 0.05) in ≥ 5% samples; (ii) coefficient of variance (CV) < 5%; (iii) methylated or unmethylated in all samples; (iv) single-nucleotide polymorphisms (SNPs) located in the assayed CpG dinucleotide [23]; and (v) did not map uniquely to the human reference genome (hg19) [24] or were on sex chromosomes [25]. Samples with > 5% undetectable probes also were excluded. BMIQ normalization was used for further type I and II probe correction [26].

DNA methylation data for validation sets were already normalized [19]. Quantile normalization was used to standardize all sample distributions.

Further, ComBat [27] was used to adjust batch effects among the three datasets using R package *sva*.

### HPV status collection

Human papillomavirus (HPV) status of the training set was based on the molecular classification, with tumor samples having more than 1000 reads from RNA sequencing aligned to HPV sequences, or with evidence of genomic integrated HPV DNA, deemed HPV-positive [28]. HPV status of GSE75537 set was based on the evidence of HPV DNA. Due to the relative high missing rate, we used multivariate imputation by chained equations (MICE) to ensure the statistical power [29].

### Preprocessing of gene expression data

Level 3 transcriptomic data of the training set were normalized by RSEM method [30]. All gene expression values were logarithmic transformed to approximate data to a normal distribution and then quantile normalized.

### Sure independence screening method

The high-dimensional microarray data (> 450,000 probes) in contrast to the small number of cases (< 320) easily leads to overfitting [31]. Regularized penalized models such as LASSO can be used to identify important variables with non-zero coefficients [32]. In this study, sure independence screening (SIS) was used based on LASSO Cox penalized regression to identify candidate CpG sites and to construct a multi-CpG-based classifier for predicting overall survival [33]. This two-stage variable screening method is more stable and reliable and was performed with R package *SIS*.

### Statistical analysis

Continuous variables were summarized as median value (range), and categorized variables were described by frequency (*n*) and proportion (%). Chi-square test was used for rate comparison. Volcano plot analysis was used to select CpG sites based on differential methylation value calculated as mean (β_tumor_) − mean (β_normal_), combined with paired Student’s *t* test *P* values. We used Spearman’s rank correlation (*r*_*s*_) to explore relationships between methylation and gene expression. Associations between characteristics and overall survival were evaluated by Cox proportional hazard models, while hazard ratio (HR) and 95% confidence interval (95% CI) were described as per 1% methylation increment.

Kaplan-Meier survival curves were drawn and compared among subgroups using log-rank tests. We predicted overall survival using the nearest neighbor method for receiver operating characteristic (ROC) curves of censored survival data [34]. Estimations of confidence intervals and *P* values of area under the curve (AUC) were based on bootstrap resampling.

VanderWeele’s mediation analysis was used to explore whether the prognostic effect of seven DNA methylation sites is mediated by affecting corresponding mRNA expression [35]. Total effect of methylation on survival (HR_total_) was decomposed to indirect effect (HR_indirect_) representing the effect of methylation mediated through affecting gene expression and direct effect (HR_direct_) indicating the effect of methylation through mediators rather than regulating the expression.

Statistical analyses were performed using R version 3.3.0 (The R Foundation). *P* values were two-sided, and *P* < 0.05 was considered statistically significant.

## Results

### Candidate CpG sites

**Fig. 1:**
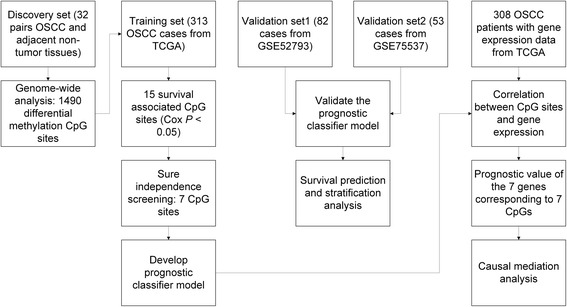
Flow chart indicating study design. We identified candidate CpG sites from 32 paired OSCC and adjacent non-tumor tissues by methylation 450k assay in the discovery set. Then, we excluded a large proportion of CpG sites that were unrelated to survival and developed prognostic scores by SIS. The seven-CpG-based classifier was validated in two independent datasets. Relationships between methylation and gene expression were also analyzed in the training dataset

**Fig. 2:**
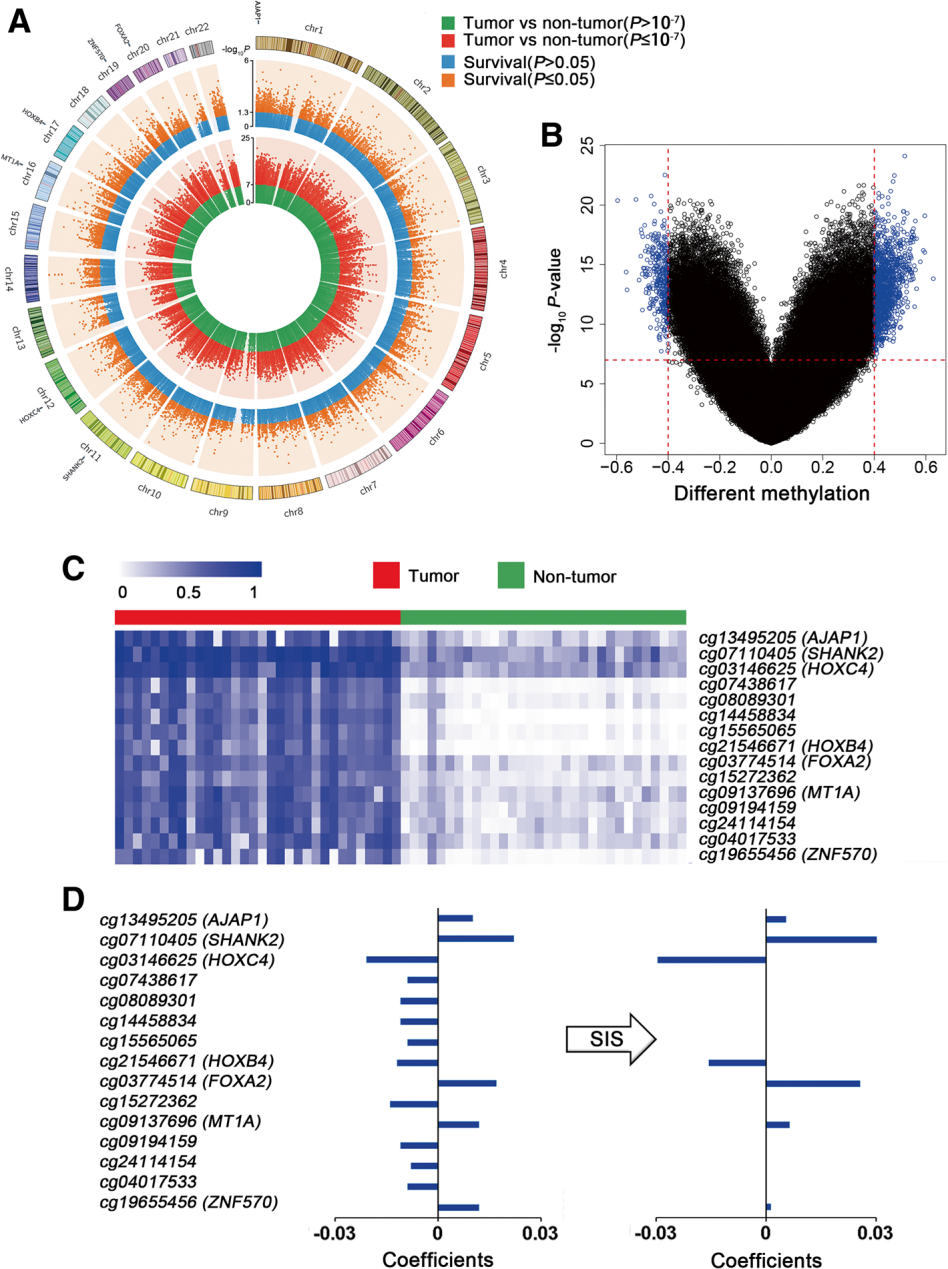
Construction of the seven-CpG-based classifier. **a** Circos plot of epigenome-wide DNA methylation CpG sites. Results are presented as *P* values ordered by genomic position, including paired *t* test of the discovery set (green and red symbols) and univariable Cox regression analysis of the training set (orange and blue symbols). **b** Volcano plot comparing CpG methylation for OSCC tumor and non-tumor tissues. A total of 1490 CpG sites had an absolute value of differential methylation of > 0.4 and a paired *t* test *P* value of < 1 × 10^−7^ (blue dots). **c** Heatmap showing methylation of 15 CpG sites in tumor tissues and adjacent non-tumor tissues. **d** Coefficients of CpG sites calculated by univariate Cox regression and sure independence screening (SIS). After SIS selection, seven probes remained non-zero coefficients

First, genome-wide differential methylation was identified from the discovery set of 32 OSCC patients which had both tumor and adjacent non-tumor tissues (Figs. 1 and 2a). The 1490 CpG sites with an absolute differential methylation of > 0.4 and paired *t* test *P* value of < 1 × 10^−7^ were identified (Fig. 2b).

Second, univariate Cox regression was used to evaluate their association with overall survival in the training set, which identified 15 CpG sites with *P* < 0.05. Further, SIS analysis was performed to further screen out a stable probe combination. Seven of the 15 candidate CpGs were identified, including cg13495205, cg07110405, cg03774514, cg09137696, cg19655456, cg03146625, and cg21546671 (Fig. 2c, Additional file 1: Table S1), mapped to *AJAP1, SHANK2, FOXA2, MT1A, ZNF570*, *HOXC4, and HOXB4*, respectively. Using coefficients generated from Cox model, we calculated a prognostic score for each patient based on individualized values of the seven genes (Fig. 2d): prognostic score_methylation_ = 0.0054 × cg13495205_*AJAP1*_ + 0.0318 × cg07110405_*SHANK2*_ + 0.0256 × cg03774514_*FOXA2*_ + 0.0063 × cg09137696_*MT1A*_ + 0.0013 × cg19655456_*ZNF570*_ − 0.0297 × cg03146625_*HOXC4*_ − 0.0157 × cg21546671_*HOXB4*_.

### Prognostic signature for OSCC patients

**Fig. 3:**
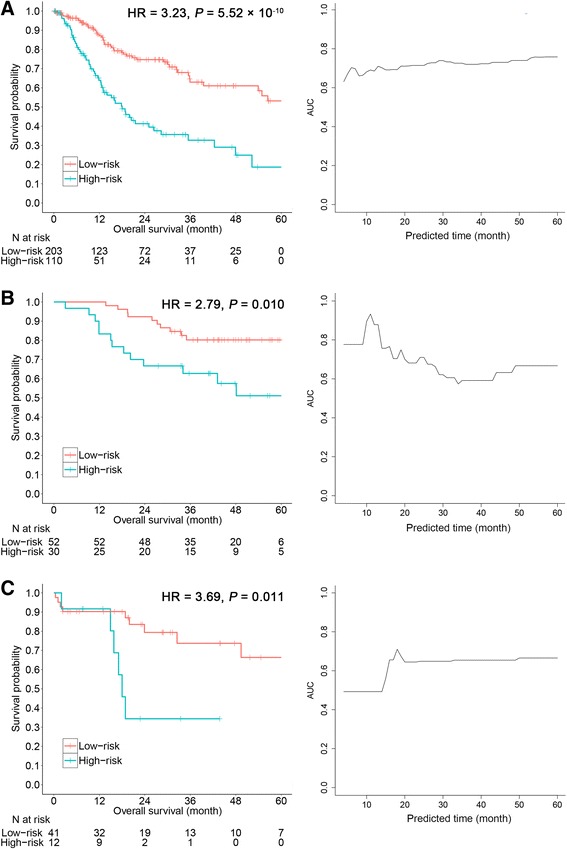
Prognostic signature and OSCC patient survival. Left panels show Kaplan-Meier survival analyses of patients, which are categorized into low-risk and high-risk groups using a cutoff value of 0.02, for the **a** training set, **b** validation set 1, and **c** validation set 2. *P* values were calculated using log-rank test, and HR indicates hazard ratio. Right panels show time-dependent ROC curves of different months used to evaluate patient survival, with risk score using the nearest neighbor method

We categorized patients into low-risk and high-risk groups using a cutoff prognostic score of 0.02, which was selected by the optimum cutoff value according to the highest *χ* ^2^ value defined by Kaplan-Meier survival analysis and log-rank test in the training set [36]. As a weighted linear combination model of seven CpG sites, higher prognostic score was significantly associated with shorter survival in the training set (HR = 3.23; 95% CI 2.18–4.77; *P* = 5.52 × 10^−10^; Fig. 3a). A significant different proportion of patients in the low-risk group (23.3%) and high-risk group (54.4%) were followed until death (*χ* ^2^ = 28.48; *P* = 9.45 × 10^−8^). Results remained significant after adjustment for HPV status, age, gender, clinical stage, smoking status, and tumor grade (HR_adjust_ = 3.14; 95% CI 1.89–5.22; *P* = 9.57 × 10^−6^; Additional file 1: Table S2).

The prognostic signature with the same classifier cutoff (0.02) were successfully validated in the two validation sets, respectively. In validation set 1, there was a 2.79-fold higher risk of death for the high-risk group compared to the low-risk group (HR = 2.79; 95% CI 1.23–6.33; *P* = 0.010; Fig. 3b). In validation set 2, there was a 3.69-fold higher risk of death for the high-risk group compared to the low-risk group (HR = 3.69; 95% CI 1.25–10.85; *P* = 0.011; Fig. 3c). After controlling for HPV status, age, gender, clinical stage, smoking status, and grade in validation set 2, the results retained statistical significance (HR_adjust_ = 2.96; 95% CI 0.53–7.26; *P* = 0.031; Additional file 1: Table S2).

Further, prediction ability of the prognostic signature was evaluated for 5-year overall survival. Time-dependent AUCs were 0.76 in the training set (95% CI 0.67–0.82; *P* < 0.001), 0.67 in validation set 1 (95% CI 0.54–0.78; *P* = 0.005), and 0.66 in validation set 2 (95% CI 0.50–0.79; *P* = 0.030) (Fig. 3a–c, right panels).

### Sensitivity analysis for the seven-CpG-based signature

**Fig. 4:**
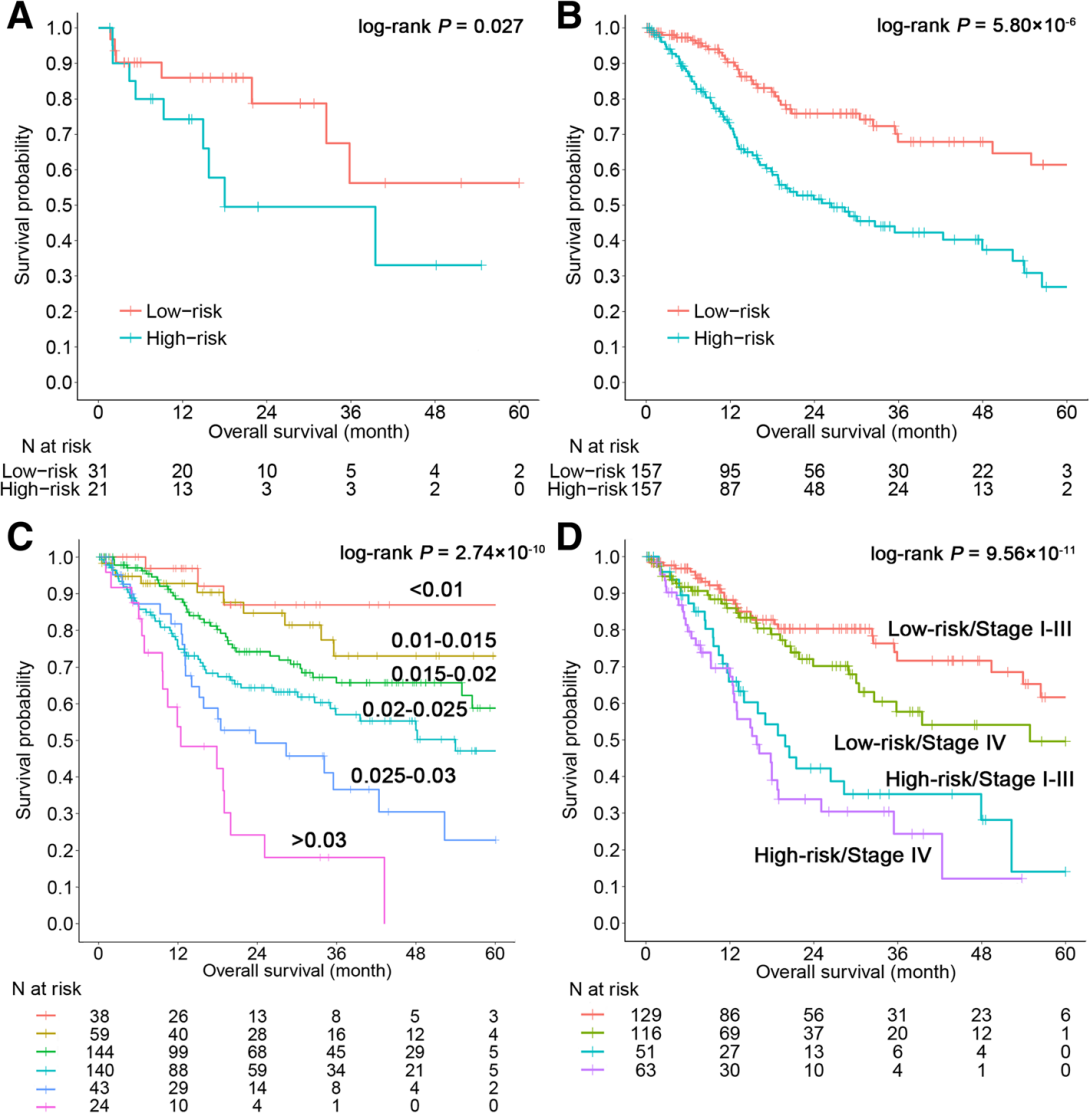
Subgroup and stratification analysis of the seven-CpG-based signature. Subgroup analysis for HPV+ cases (**a**) and HPV− cases (**b**) in the imputed combined dataset. **c** Kaplan-Meier curves plotting overall survival of the combined three datasets for respective prognostic score categories. **d** Subgroup analysis with clinical stage of the combined training set and validation set 2

Due to the small sample size of HPV-positive cases, the training set and validation set 2 were merged to explore the relationship between the prognostic score and HPV status. Using multiple linear regression adjusted for age, gender, stage, smoking status, and grade, the score was not associated with HPV status (*β* = − 0.01; 95% CI − 0.23–0.20; *P* = 0.898). In the subgroup analysis stratified by HPV status, the seven-CpG-based signature was significant both within HPV-positive cases (HR = 3.33; 95% CI 1.07–10.37; *P* = 0.027; Fig. 4a) and HPV-negative cases (HR = 2.65; 95% CI 1.73–4.04; *P* = 5.80 × 10^−6^; Fig. 4b).

Using the data merged of training and validation sets, as shown in Fig. 4c, prognostic score showed a stronger association with overall survival (log-rank *P* = 2.74 × 10^−10^). Stratified analyses by clinical characteristics (clinical stage, age, gender, smoking status, and grade) retained statistical significance (Fig. 4d and Additional file 1: Figure S2).

### Relationship of CpG methylation, gene expression, and prognosis

**Fig. 5:**
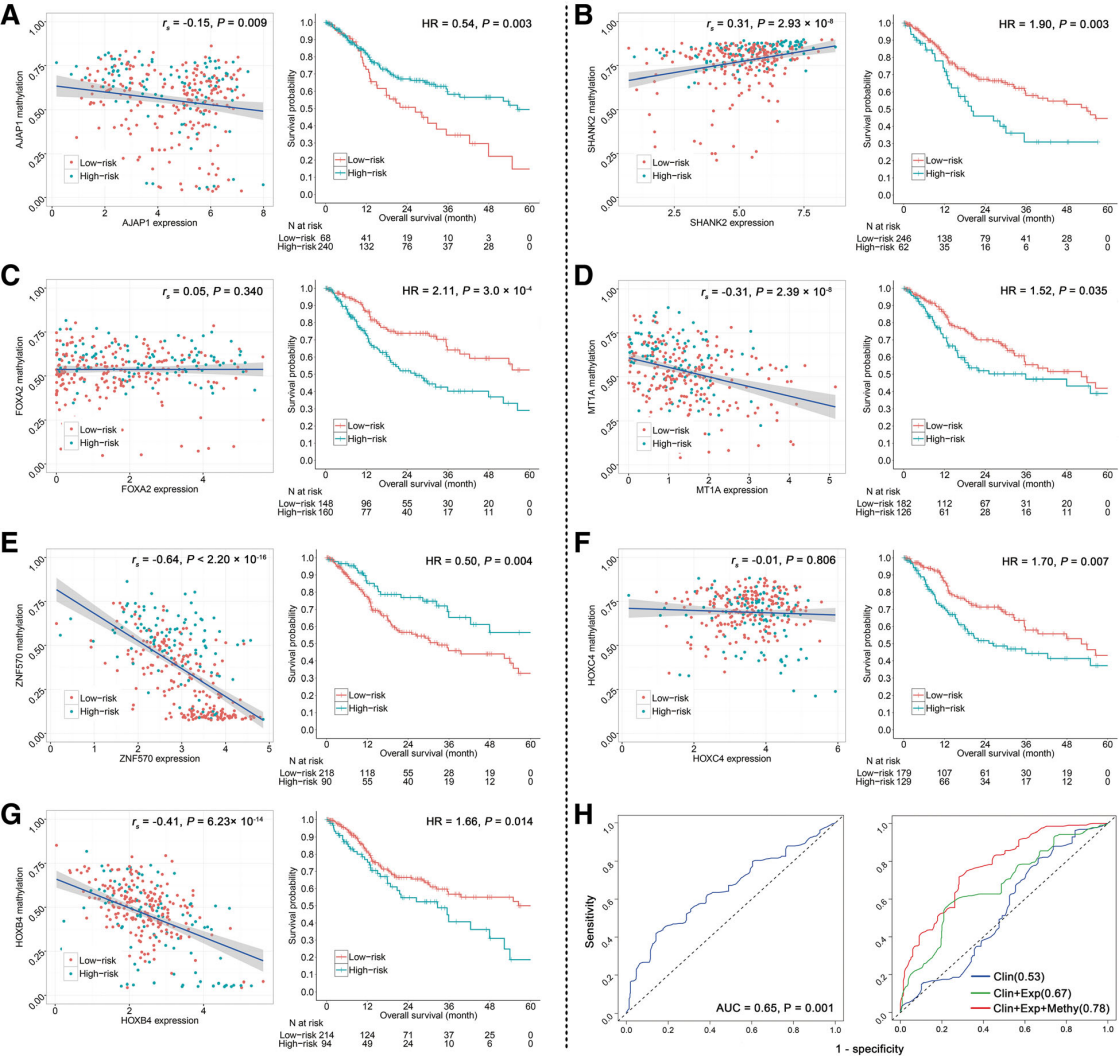
Association between gene expression and methylation. Left panels show correlation of **a** *AJAP1*, **b** *SHANK2*, **c** *FOXA2*, **d** *MT1A*, **e** *ZNF570*, **f** *HOXC4*, and **g** *HOXB4* expression (*X*-axis) with methylation (*Y*-axis). Right panels show Kaplan-Meier survival plots of gene expression from the TCGA cohort. HR indicates hazard ratio. Correlation coefficients and hypothesis tests were based on Spearman rank correlation tests. Patients were categorized into high-risk and low-risk groups by an optimum cutoff point according to the highest *χ* ^2^ value. **h** ROC curves for expression of the seven genes (left) and combinations of different types of data (right), including clinical characteristics (Clin), gene expression (Exp), and methylation (Methy)

Methylation and expression quantitative trait loci (meQTL) relationship for the seven CpG sites was performed in the training set. Expression and methylation data were both available in 308 cases of training set (Table 2). Methylation level of CpG sites at the promoter region and 1st exon region was moderately correlated with the corresponding gene expression for *AJAP1* (*r*_*s*_ = − 0.15; *P* = 0.009), *HOXB4* (*r*_*s*_ = − 0.41; *P* = 6.23 × 10^−14^), *MT1A* (*r*_*s*_ = − 0.31; *P* = 2.39 × 10^−8^), *ZNF570* (*r*_*s*_ = − 0.64; *P* < 2.20 × 10^−16^), and *SHANK2* (*r*_*s*_ = 0.31; *P* = 2.93 × 10^−8^). Methylation of the other two CpG sites located in the gene body of *HOXC4* (*r*_*s*_ = − 0.01; *P* = 0.806) and *FOXA2* (*r*_*s*_ = 0.05; *P* = 0.340) was not observed any correlation with the gene expression (Fig. 5a–g, left panels). These genes’ expression was also significantly associated with patient’s overall survival (Fig. 5a–g, right panels).

**Table 2:** Clinical characteristics of the training set with both methylation and mRNA data

Characteristic	Subset of training set with both DNA methylation and mRNA expression data (*N* = 308)
Censor rate	66.9%
Age, median years (range)	61.0 (19–90)
Gender, *n* (%)	
Male	206 (66.9)
Female	102 (33.1)
Smoking status, *n* (%)	
Never	84 (27.3)
Current/former	216 (70.1)
NA	9 (2.6)
Race, *n* (%)	
White	267 (86.7)
Black or African American	20 (6.5)
Asian	10 (3.2)
American Indian or Alaska Native	1 (0.3)
NA	10 (3.2)
HPV status, *n* (%)	
Positive	13 (4.2)
Negative	175 (56.8)
NA	120 (39.0)
TNM stage, *n* (%)	
I	12 (3.9)
II	75 (24.4)
III	64 (20.8)
IV	150 (48.7)
NA	7 (2.3)
Grade, *n* (%)	
G1	48 (15.6)
G2	193 (62.7)
G3	63 (20.5)
NA	4 (1.3)

*NA* not available

Prognostic score using the expression of seven genes was also calculated (score_expression_ = − 0.115 × *AJAP1* + 0.089 × *SHANK2* + 0.147 × *FOXA2* + 0.111 × *MT1A* − 0.173 × *ZNF570* + 0.030 × *HOXC4* + 0.789 × *HOXB4*), which was significantly associated with the prognosis (dichotomized by median, HR = 2.20; 95% CI 1.47–3.29; *P* = 1.22 × 10^−4^). After adjustment for HPV status, age, gender, clinical stage, smoking status, and grade, the result was still significant (HR = 3.41; 95% CI 1.98–5.89; *P* = 1.07 × 10^−5^) (Additional file 1: Figure S3). In addition, it effectively predicted 5-year survival (AUC = 0.65; 95% CI 0.54–0.72; *P* = 0.001) (Fig. 5h, left panel).

Combination of clinical information, expression, and methylation data (AUC = 0.78) showed a superior prediction ability in comparison to the model using clinical data only (AUC = 0.53) or clinical and expression data (AUC = 0.67) (Fig. 5h, right panel).

**Fig. 6:**
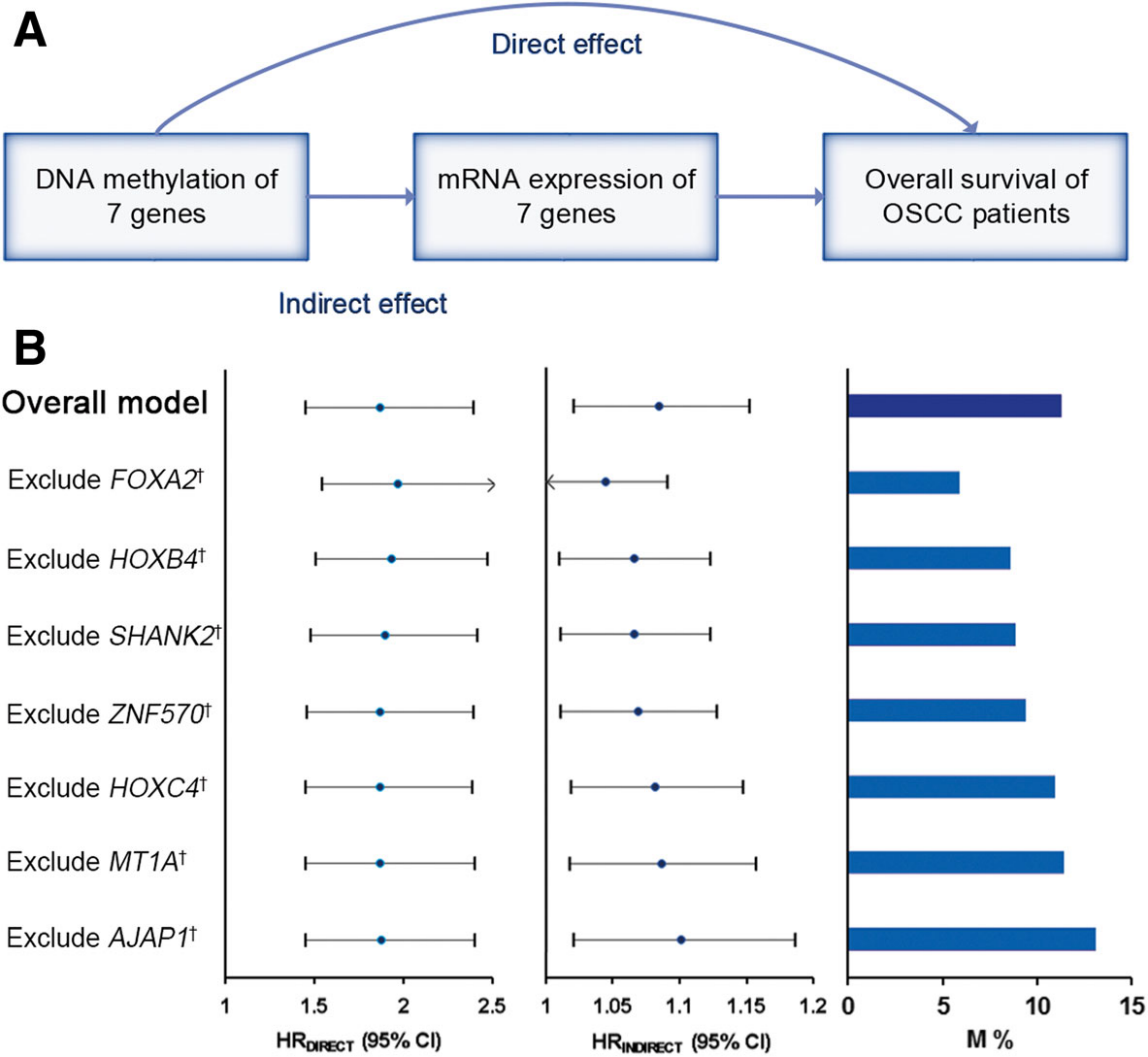
Mediation analysis for methylation prognostic signature through mRNA expression. **a** Diagram of mediation model. **b** Methylation signature from the seven CpG sites was treated as “exposure”; mediator was the linear combination of the corresponding seven genes’ expression level (score_expression_) (Overall model). Total prognostic effect in hazard ratio (HR) were described as direct effect (HR_direct_), indirect effect (HR_indirect_), corresponding 95% confidence interval (95% CI), and the proportion of effect mediated (M%). Further, sensitivity analyses were performed by excluding each gene from score_expression_, respectively, which retained statistical significance for mediation effect

Furthermore, VanderWeele’s mediation analysis was used to explore the underlying mediation pathway of methylation, mRNA expression, and overall survival (Fig. 6a). Score_expression_, the linear combination of seven genes’ mRNA expression, was treated as mediator in the overall mediation model. The prognostic effect of methylation signature was significantly mediated through affecting their mRNA expression (HR_indirect_ = 1.08; 95% CI 1.02–1.15; *P* = 0.008; proportion mediated, 11.27%). Sensitivity analysis by excluding each gene expression from score_expression_ retained statistical significance (Fig. 6b).

## Discussion

Cancer involves a complex regulatory network, integrating multiple biomarkers into an aggregated model could improve prognostic value compared with single biomarker [37]. The biomarkers discovery for OSCC have been reported in several studies [38, 39], but few of them used more than two datasets or explore the biomarkers across different omics. In this study, we developed an OSCC prognostic classifier model that includes seven CpG sites and validated the model using two independent external datasets. Results show that the prognostic signature was significantly associated with OSCC patient overall survival and had certain prediction abilities in the three datasets tested. OSCC patients with higher prognostic scores tended to have poorer clinical outcomes. Further, the gene expression of the corresponding CpG sites were also associated with overall survival. The integrated model of methylation and expression could add prognostic predictive value based on the clinical information (e.g., HPV status, age, clinical stage, and grade).

We used a three-step selection method to screen out significant biomarkers from more than 320,000 CpG sites after quality control. Differential methylation analysis using paired tissue data as the first step excluded 99.5% of probes. To exclude probes unrelated to survival, we evaluated their prognostic values by univariable Cox regression as the second step. However, the Cox model is not suitable for accurate modeling due to the low sample size/variable ratio and unstable variable combination [40]. To overcome the problem, SIS, a method based on a LASSO penalized model, was used to select a more stable and reliable set of CpG sites for further modeling. It first screened all included variables and discarded the irrelevant features with weak correlation to overall survival, then applied LASSO to estimate sensitivity from the selected genomic instability data [41].

The Cancer Genetics Web [42] suggests that research on OSCC biomarkers is still not comprehensive enough. Our study provides seven significant prognostic genes at the epigenetic and transcriptomic levels. Among the seven genes corresponding to candidate CpG sites, six have been reported as cancer-related genes. *AJAP1*, a novel tumor suppressor gene, is associated with survival in esophageal squamous cell carcinoma [43], hepatocellular carcinoma [44], and glioma [45]. Demethylation of hypermethylated *AJAP1* reactivates its mRNA expression [43]. *SHANK2* might cooperate with *EMS1* to encode cytoskeleton-associated proteins implicated in tumor cell motility and invasiveness in OSCC [46]. It is also hypermethylated in prostate cancer tissues compared with paired non-tumor tissues [47]. *FOXA2* is implicated in increased relapses and risk prognostic value in triple-negative/basal-like breast tumors [48]. Conversely, *FOXA2* also is downregulated in lung cancer through epigenetic silencing of hypermethylation [49]. Further experiments are needed to verify the function of *FOXA2* in OSCC. *MT1A*, which regulates cell growth and differentiation, has been described as a hypermethylated CpG biomarker for OSCC [50]. *MT1A* overexpression is also associated with HNSCC [51]. *HOXC4* and *HOXB4* are hypermethylated and downregulated in high-risk groups, and hypermethylation of *HOXB4* is inversely correlated with decreased expression as an epigenetic biomarker for OSCC [52]. In addition, *HOXC4* triggers similar molecular alterations as *HOXB4* [53] and also is involved in some cancers [54, 55]. *ZNF570* belongs to the large zinc finger gene family, which has been reported that is useful in the detection of HNSCC [56]. Although this gene’s function is still not known well, we measured a strong negative correlation of *ZNF570* methylation and expression, both of which were significant in patient prognosis. Therefore, additional experiments are required; *ZNF570* may represent a novel OSCC biomarker.

In addition to DNA methylation, mRNA expression levels of seven genes also affect prognosis significantly. Around 11% of methylation prognostic effect is mediated through affecting corresponding gene expression. Interestingly, most of the methylation’s effect may act, beyond affecting expression, but gene function [57], which warrants further functional experiments.

However, our study has some limitations. First, baseline information for GEO validation set 1 is unavailable, so multivariable analysis could not be made in the validation phase for this dataset. Second, due to the small sample size of some groups in the stratification analysis, like the HPV-positive cases, the results should be taken with caution since the sample size is insufficient. Third, further studies are needed to verify the biological function of some genes.

## Conclusions

This study suggests that the developed seven-CpG-based signature coupled with gene expression is a useful and practical tool to improve prognostic value and survival prediction of OSCC, indicating it may have new applications for appropriate clinical adjuvant trials. Future studies including these molecular methylation and/or gene expression biomarkers, HPV status, age, other clinical characteristics, and different therapy effects will be useful for developing future personalized treatments.

## Additional file

Additional file 1: Table S1. Annotation for seven CpG sites selected by SIS. Table S2 Cox regression analysis of clinical characteristics and risk scores. Figure S1. Boxplot depicting beta-values of seven CpG sites after ComBat processing in training and validation datasets. Figure S2. Kaplan-Meier survival analyses of patients subgrouped by (A) age divided by median value (60 years), (B) gender, (C) smoking status, or (D) grade. Figure S3. Kaplan-Meier survival analyses of the gene expression prognostic score. Low-risk and high-risk patients were divided by the median value. (DOCX 832 kb)

## Abbreviations

95% CI: 95% confidence interval
AUC: Area under the curve
CV: Coefficient of variance
GDC: Genomic Data Commons
GEO: Gene Expression Omnibus
HNSCC: Head and neck squamous cell carcinoma
HPV: Human papillomavirus
HR: Hazard ratio
MICE: Multivariate imputation by chained equations
OSCC: Oral squamous cell carcinoma
ROC: Receiver operating
SIS: Sure independence screening
SNP: Single-nucleotide polymorphism
TCGA: The Cancer Genome Atlas
