# Supplementary material for: Seven-CpG-based prognostic signature coupled with gene expression predicts survival of oral squamous cell carcinoma
Source: Clin Epigenetics. 2017 Aug 24;9:88. doi: 10.1186/s13148-017-0392-9 (PMC5571486; doi:10.1186/s13148-017-0392-9)
Supplement: Additional file 1: Table S1. — Annotation for seven CpG sites selected by SIS. Table S2 Cox regression analysis of clinical characteristics and risk scores. Figure S1. Boxplot depicting beta-values of seven CpG sites after ComBat processing in training and validation datasets. Figure S2. Kaplan-Meier survival analyses of patients subgrouped by (A) age divided by median value (60 years), (B) gender, (C) smoking status, or (D) grade. Figure S3. Kaplan-Meier survival analyses of the gene expression prognostic score. Low-risk and high-risk patients were divided by the median value. (DOCX 832 kb) [file 13148_2017_392_MOESM1_ESM.docx]

## Prognostic signature from DNA methylation and corresponding gene expression predicts survival of oral squamous cell carcinoma

**Running head:** DNA methylation, gene expression and OSCC patient survival

Sipeng Shen, Guanrong Wang Qianwen Shi, Ruyang Zhang, Yang Zhao, Yongyue Wei, Feng Chen, David C. Christiani

**Supplementary Tables and Figures**

**Table S1. Annotation for seven CpG sites selected by SIS**

| CpG site | CHR | Position | UCSC_RefGene_Name | Number of CpGs  of this gene | UCSC_RefGene_Group | Relation_to_UCSC_CpG_Island |
| --- | --- | --- | --- | --- | --- | --- |
| cg13495205 | 1 | 4714033 | *AJAP1* | 70 | TSS1500 | Island |
| cg07110405 | 11 | 70917533 | *SHANK2* | 238 | 5'UTR |  |
| cg03774514 | 20 | 22563702 | *FOXA2* | 20 | Body | Island |
| cg09137696 | 16 | 56672415 | *MT1A* | 9 | TSS200 | Island |
| cg19655456 | 19 | 37959961 | *ZNF570* | 14 | TSS200 | Island |
| cg03146625 | 12 | 54448729 | *HOXC4* | 111 | Body | S_Shore |
| cg21546671 | 17 | 46655387 | *HOXB4* | 27 | 1^st^ exon | Island |

**Table S2. Cox regression analysis of clinical characteristics and risk scores**

| Characteristics | Training set | | | |  | Validation set 2 | | | |
| --- | --- | --- | --- | --- | --- | --- | --- | --- | --- |
|  | Univariable | | Multivariable | |  | Univariable | | Multivariable | |
|  | HR (95% CI) | *P* | HR (95% CI) | *P* |  | HR (95% CI) | *P* | HR (95% CI) | *P* |
| High risk score | 3.23 (2.18-4.77) | 5.52×10^-10^ | 3.15 (2.08-4.77) | 5.97×10^-8^ |  | 3.69 (1.25-10.85) | 0.011 | 2.96 (0.53–7.26) | 0.031 |
| HPV (positive) | 0.90 (0.39-2.09) | 0.816 | 0.70 (0.29-1.69) | 0.429 |  | 1.01 (0.35-2.87) | 0.981 | 0.62 (0.27-9.44) | 0.599 |
| Age (per year) | 1.01 (0.99-1.02) | 0.096 | 1.01 (0.99-1.03) | 0.108 |  | 1.02 (0.98-1.06) | 0.180 | 1.04 (1.00-1.10) | 0.047 |
| Gender (female) | 1.03 (0.69-1.53) | 0.874 | 1.05 (0.65-1.70) | 0.821 |  | 2.71 (0.59-12.41) | 0.197 | 1.35 (0.21-8.64) | 0.749 |
| Clinical stage (per stage) | 1.19 (0.96-1.48) | 0.095 | 1.17 (0.94-1.45) | 0.158 |  | 1.58 (0.97-2.59) | 0.064 | 1.97 (1.07-3.63) | 0.028 |
| Smoking status (smoker) | 1.30 (0.82-2.06) | 0.262 | 1.18 (0.72-1.92) | 0.498 |  | 2.04 (0.73-5.72) | 0.172 | 1.36 (0.42-4.42) | 0.600 |
| Grade (per grade) | 1.41 (1.03-1.92) | 0.028 | 1.79 (1.21-2.64) | 0.003 |  | 2.32 (1.04-5.20) | 0.039 | 2.44 (0.89-6.72) | 0.082 |

Baseline information of validation set 1 is unavailable in GEO.

**
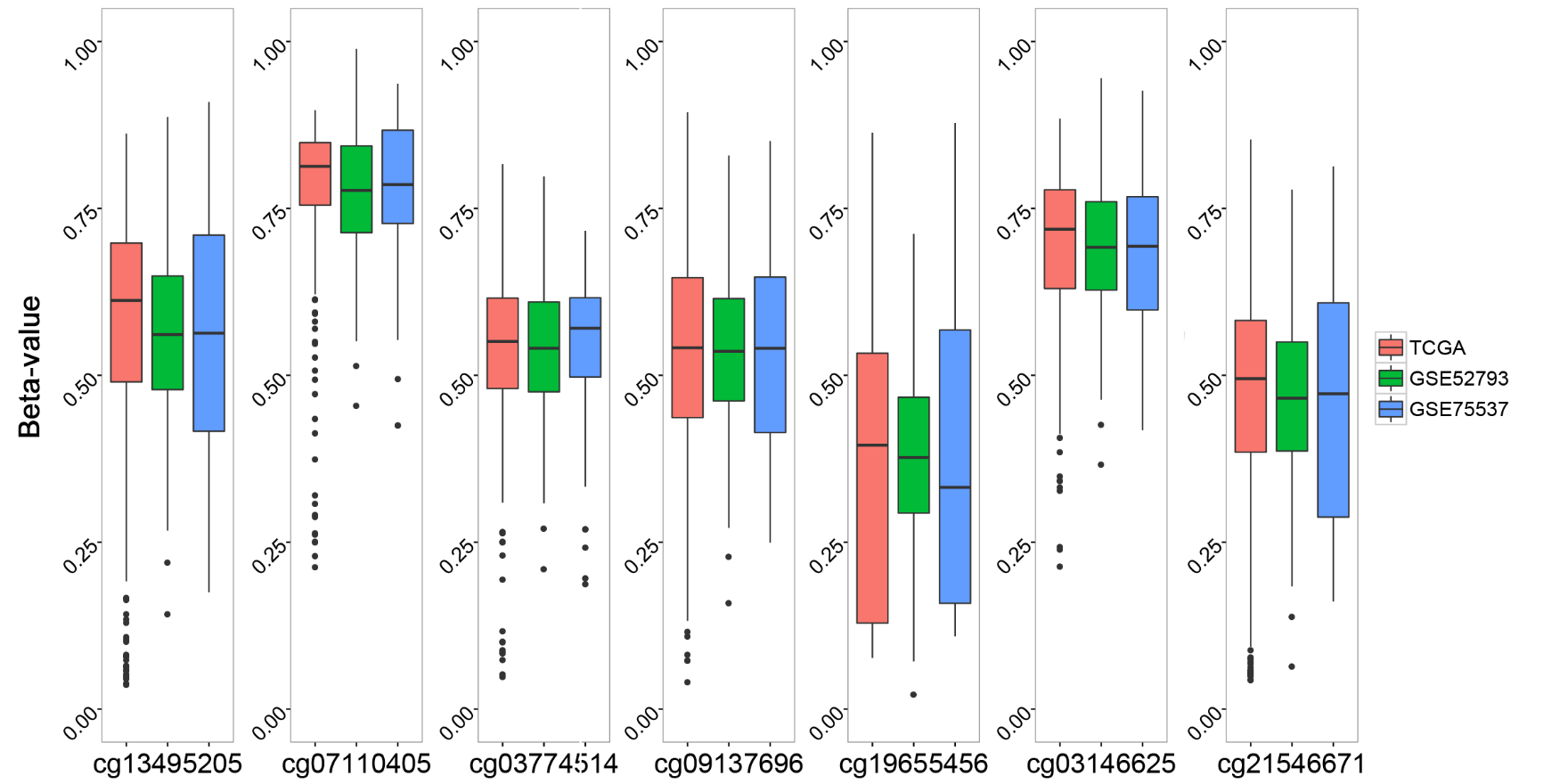
**

**Figure S1.** Boxplot depicting beta-values of seven CpG sites after ComBat processing in training and validation datasets.

**
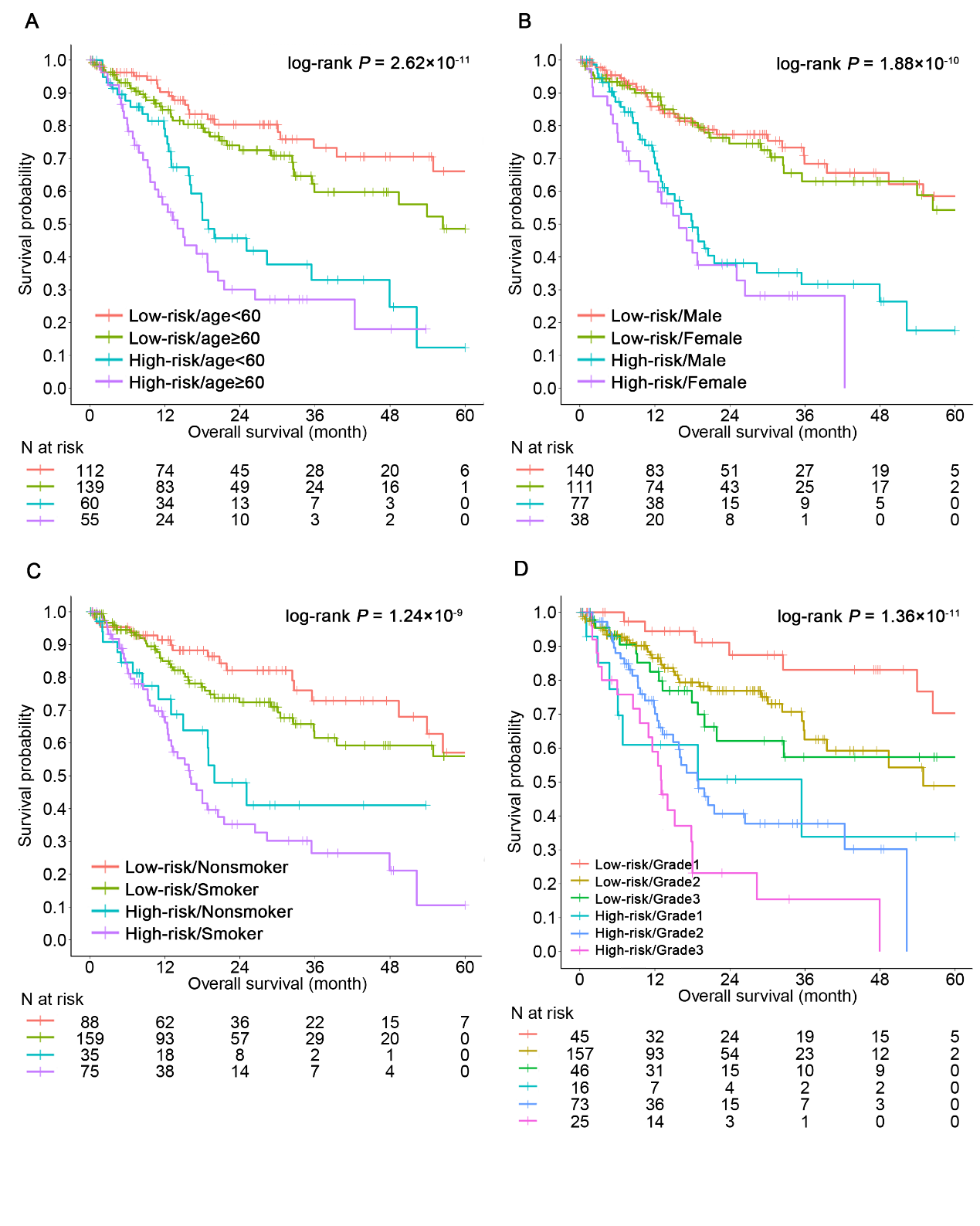
**

**Figure S2.** Kaplan-Meier survival analyses of patients subgrouped by (**A**) age divided by median value (60 years), (**B**) gender, (**C**) smoking status, or (**D**) grade.


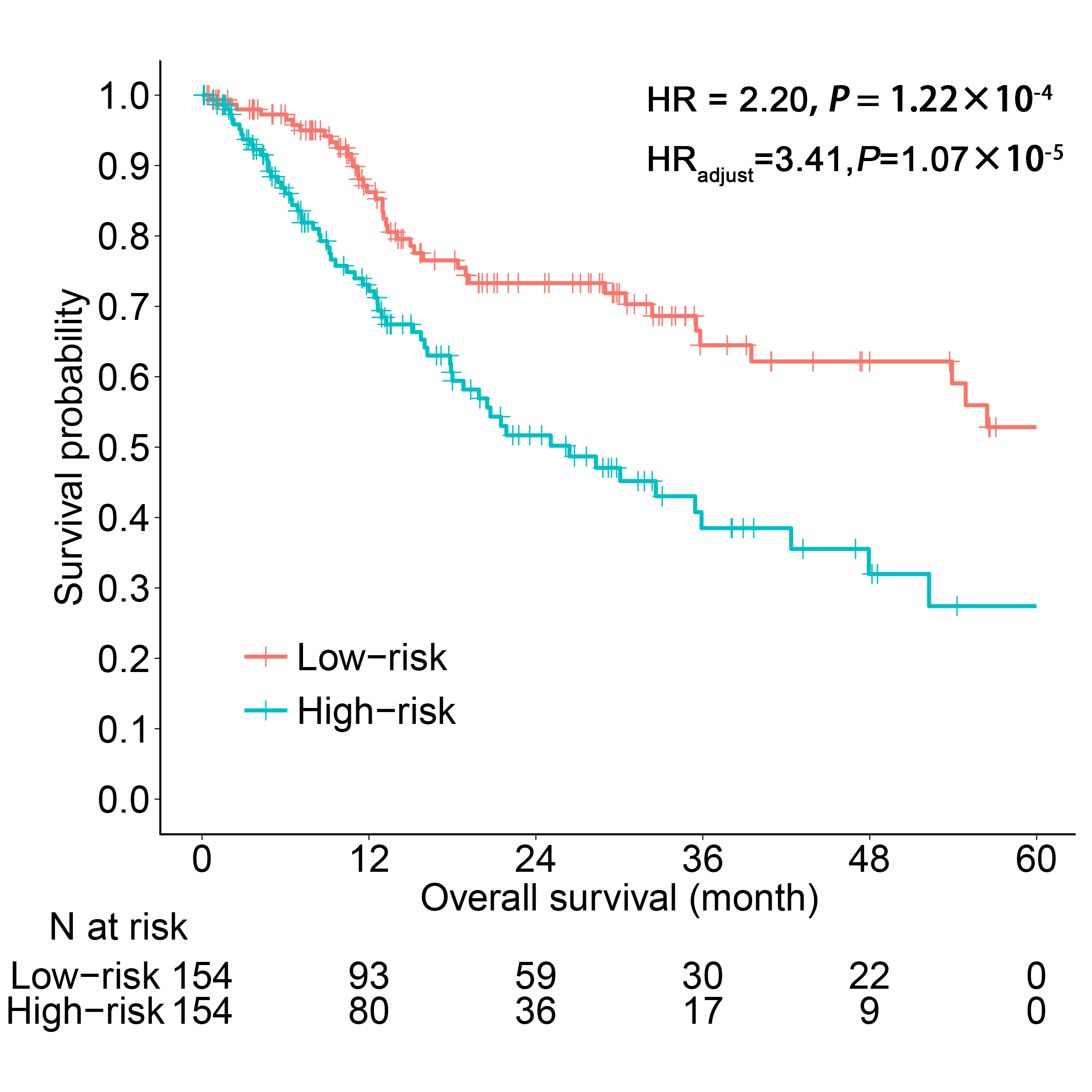


**Figure S3.** Kaplan-Meier survival analyses of the gene expression prognostic score. Low-risk and high-risk patients were divided by the median value.
